# Technology of plant factory for vegetable crop speed breeding

**DOI:** 10.3389/fpls.2024.1414860

**Published:** 2024-07-11

**Authors:** Rui He, Jun Ju, Kaizhe Liu, Jiali Song, Shuchang Zhang, Minggui Zhang, Youzhi Hu, Xiaojuan Liu, Yamin Li, Houcheng Liu

**Affiliations:** College of Horticulture, South China Agricultural University, Guangzhou, China

**Keywords:** vegetable, speed breeding, plant factory, smart breeding, breeding cycle

## Abstract

Sustaining crop production and food security are threatened by a burgeoning world population and adverse environmental conditions. Traditional breeding methods for vegetable crops are time-consuming, laborious, and untargeted, often taking several years to develop new and improved varieties. The challenges faced by a long breeding cycle need to be overcome. The speed breeding (SB) approach is broadly employed in crop breeding, which greatly shortens breeding cycles and facilities plant growth to obtain new, better-adapted crop varieties as quickly as possible. Potential opportunities are offered by SB in plant factories, where optimal photoperiod, light quality, light intensity, temperature, CO_2_ concentration, and nutrients are precisely manipulated to enhance the growth of horticultural vegetable crops, holding promise to surmount the long-standing problem of lengthy crop breeding cycles. Additionally, integrated with other breeding technologies, such as genome editing, genomic selection, and high-throughput genotyping, SB in plant factories has emerged as a smart and promising platform to hasten generation turnover and enhance the efficiency of breeding in vegetable crops. This review considers the pivotal opportunities and challenges of SB in plant factories, aiming to accelerate plant generation turnover and improve vegetable crops with precision and efficiency.

## 1 Introduction

The current trajectory for crop productivity needs to produce 60% more food to sustainably nourish a burgeoning world population of 10 billion by 2050; therefore, there is an urgent need for higher-yielding varieties and accelerating crop production (Pardey et al., 2014). The ever-increasing human population, urbanization, declining agricultural lands, the rising frequency of destructive weather events, and the high incidence of pests and diseases pose grim threats to global food security (Shrivastava and Kumar, 2015; Alotaibi, 2023). In particular, during the coronavirus disease 2019 (COVID-19) pandemic era, improving crop productivity to alleviate food scarcity problems and ensuring food and nutritional security through modern breeding technologies are crucial for plant breeders and scientists.

## 2 The demand for faster vegetable crop breeding

The length of the breeding cycle is a major issue for crop genetic improvement (Saxena et al., 2017; Wanga et al., 2021). The development of genetically stable homozygous or novel cultivars with market-preferred traits of crops is time-consuming, which generally takes up to a decade or more via conventional breeding methodologies. Hence, shortening crop breeding cycles through promoting rapid plant growth and early flowering has greatly attracted the attention of plant breeders and researchers worldwide for crop production and productivity improvement (Watson et al., 2018; Bhatta et al., 2021; Guo et al., 2022; Liu et al., 2022). Efficient strategies are being employed to greatly reduce generation time including shuttle breeding, doubled haploid, targeting-induced local lesions in genomes (TILLING), and genome editing technologies (**Table 1**). However, these molecular and conventional breeding approaches cannot satisfy the ever-increasing demands for agricultural production and more available breeding approaches need to be explored.

**Table 1:** Strategies to speed up the breeding process of crops.

Strategies	Basics of methods	Generation per year	Limitations	Reference
**Shuttle-breeding**	Growing crop material in alternative suitable locations.	2–3	⋄ Not reliable ⋄ High rate of loss of material ⋄ Reducing genetic variance	Ortiz et al., 2007
**Double haploid (DH)**	Facilitating the development of entirely homozygous lines in two generations.	3–4	⋄ Genotype dependence ⋄ Requiring special skills and labor ⋄ High cost	Germanà, 2011
***In vitro* culture**	Using nutritive culture media and controlled aseptic conditions for the growth of plant cells, tissues, organs, or immature embryos.	12	⋄ Specific protocol for each species ⋄ High cost ⋄ Requiring specialized staff ⋄ Labor intensive	Germanà, 2011
**Targeting induced local lesions in genomes**	A reverse genetics approach for high-throughput discovery of induced mutations in the desired gene(s) from a mutant population developed through mutagenesis.	Not available	⋄ High screening costs ⋄ Large insertions or deletions are difficult to detect ⋄ Rely on known genome ⋄ Dependence on random mutagenesis	Mba, 2013; Chen et al., 2014
**Transgenesis or genetic editing**	Transgenesis involves the introduction of foreign genes into an organism’s genome, while genetic editing allows for precise modifications to be made to the organism’s existing DNA sequence.	Not available	⋄ Political and social issues ⋄ The lack of regulatory hurdle ⋄ Specific protocol for each species ⋄ Genotype dependence and requiring specialized staff	Zhan et al., 2021
**Single seed descent (SSD)**	Sampling one seed of each plant in a segregating population and continue this process until the desired level of inbreeding has been achieved.	Not available	⋄ More inferior progenies ⋄ Risks of losing desirable genes ⋄ Requiring appropriate facilities	Kigoni et al., 2023
**Speed breeding (SB)**	Using optimal day length, light intensity, light quality, and temperature to stimulate flowering and seed production.	4–6	⋄ Genotype dependence ⋄ High establishment cost ⋄ High energy consumption	Watson et al., 2018

In recent years, a system called speed breeding (SB) has been in the spotlight (Watson et al., 2018). It allows plant breeders to drastically shorten crop life cycles to produce more generations per year by optimized photoperiod, light quality, light intensity, day/night temperature and humidity, and CO_2_ concentration under controlled environments. This approach allows multiple generations of wheat, barley, rice, and legumes to be produced within a single year, significantly shortening the development cycles (Watson et al., 2018). Vegetable crops are integral to dietary diversity, nutritional sustenance, and economic development. While significant progress has been achieved in vegetable breeding, there remains a pressing need to expedite the development of green, environmentally resilient cultivars characterized by high yield and quality to meet the demands of the global population in a timely manner. Previous extensive reviews have summarized the methods, applications, potential advantages, and key challenges of SB techniques that can enhance crop quality and yield to meet the growing demand for food and ensure global food security. Certain reviews also focused on exploring the integration of SB with new breeding techniques (AI in crop breeding, genomics-assisted breeding, haplotype-based breeding, etc.) to efficiently and rapidly produce stable lines for both basic and applied research purposes. The current review mainly discusses the key technologies used in plant factories for efficient vegetable breeding and highlights the potential advantages of SB in plant factories as a smart and promising platform to hasten generation turnover.

## 3 Speed breeding technology utilizing plant factories

**Figure 1:**
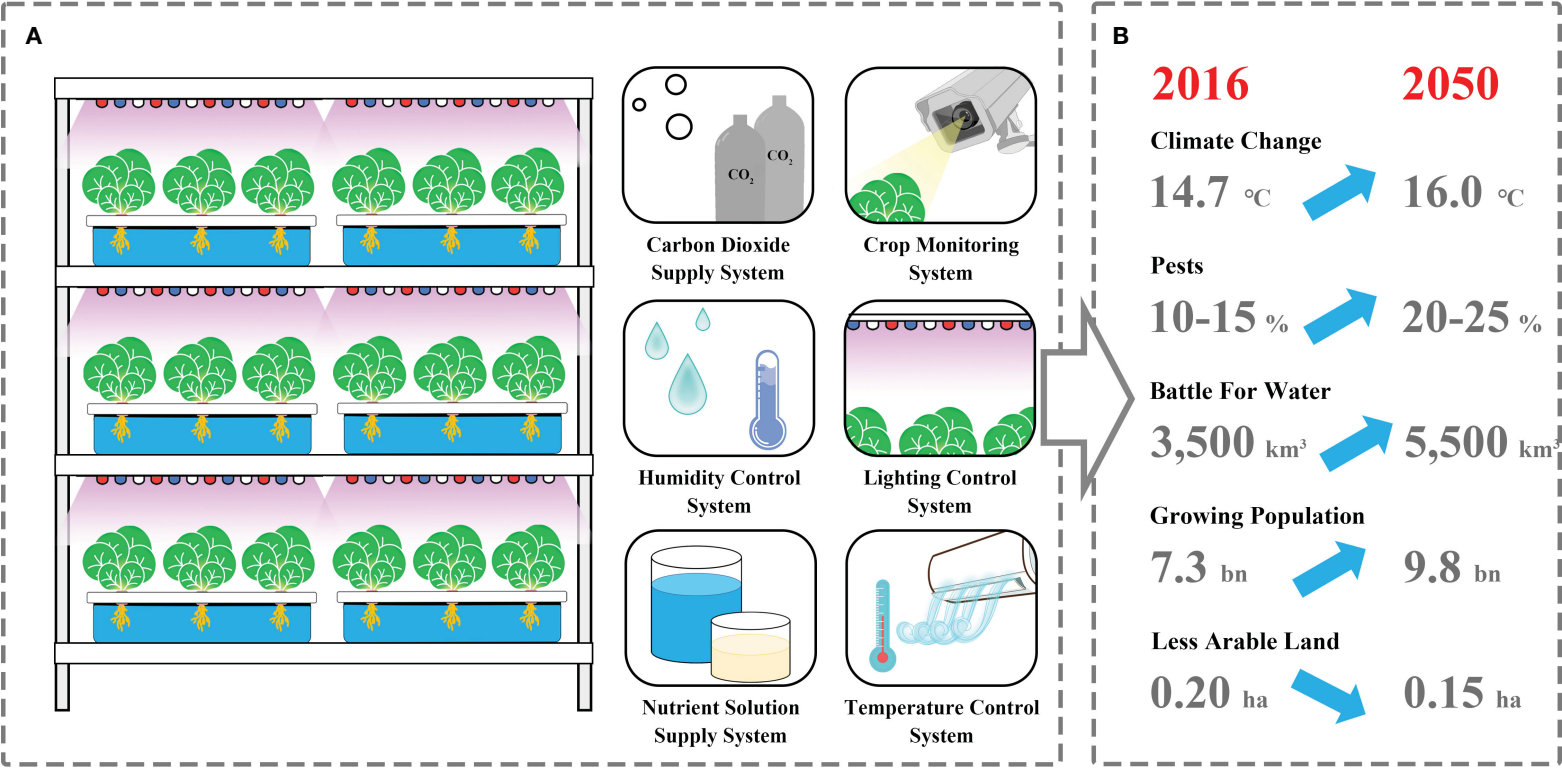
The primary structural components of a plant factory **(A)** are advantageous for coping with adverse factors **(B)** while simultaneously fulfilling the requirements for rapid crop breeding.

Plant factories are considered as multilayer closed plant production systems in which light, temperature, humidity, CO_2_, and nutrients are precisely manipulated through various sensors and artificial intelligence systems, allowing the precise control of physiological and developmental processes in plants (Kozai and Niu, 2015a) (**Figure 1A**). Crop production in plant factories can overcome many limitations of pathogens, pests, soil conditions, or climate change, allowing year-round cultivation at any location around the globe (**Figure 1B**). SB in plant factories paves a way to access four or more generations per year by providing optimal light and temperature, accelerating biological processes for rapid generation advancement (Ghosh et al., 2018; Watson et al., 2018). The shift from the vegetative to the flowering stage is one of the most crucial developmental milestones in the plant life cycle. Accelerating plant growth and development and promoting early flowering in plant factories, where various environmental factors, namely, photoperiod, temperature, and light quality and intensity, were regulated precisely and efficiently, are conducive to shortening the breeding cycles of vegetable crops Ghosh et al., 2018; Watson et al., 2018. Vegetables exhibiting short growth cycles and sensitivity to environmental cues are ideal candidates for SB, such as tomato, cucumbers, hot pepper, cabbage, spinach, and mustard greens. Their suitability for this method lies in their swift life cycles, enabling rapid generational turnover and efficient assessment of breeding characteristics. Furthermore, their adaptability to controlled environments permits precise adjustment of factors such as light, temperature, and humidity, facilitating accelerated vegetable growth rates.

## 4 Opportunities for vegetable crops speed breeding in plant factories

### 4.1 Photoperiod

Photoperiod is a pivotal component of light environmental factors that has a prominent influence on vegetative and phenological development and physiological reactions. The effect of photoperiod on flowering time and seeding of plants varies on crop genotype or cultivar. Plants are classified into long-day, short-day, or day-neutral plants based on their sensitivity response to photoperiod (Jackson, 2009). For long-day plants, the time of flowering was frequently accelerated with a prolonged photoperiod, while in short-day plants, flowering is triggered when photoperiod becomes shorter than their critical photoperiod, or night time becomes longer than the critical night period (Jackson, 2009).

SB is an approach relying on prolonging the photoperiod, that is, providing a longer duration of lighted period to hasten plant growth and accelerate the plant life cycle (Watson et al., 2018). Efforts toward SB of vegetable crops mainly focus on accelerating the transition from vegetative to reproductive development. Optimum photoperiod is critical and responsible for vegetative and reproductive growth. Thus, manipulation of the photoperiod regime has been well applied in many plant species to stimulate early flowering and seeding and shorten breeding cycles, such as in wheat, canola, and hot pepper (**Table 2**). For long-day plants, although the days of flowering seem to decrease linearly with prolonging the photoperiod, some stress and damage such as foliar chlorosis, leaf burn, flower abortion, wilting, and mortality, caused by an artificially longer photoperiod, should not be neglected (Takahashi and Badger, 2011; Omar, 2020; Mitache et al., 2023). Short-day plants initiate flowering when the photoperiod is shorter than a critical day length, but to date, the sensitive period of short-day crops to flower is less unknown. Therefore, the optimum light/dark ratio that accelerates the plant growth of various stress-free crops still needs more exploration.

**Table 2:** Effect of light photoperiod on crop speed breeding.

Plant species	Light photoperiod	Other experimental details	Influence on speed breeding generations		Reference
			Effects	Generations/year	
Spring wheat (*Triticum aestivum*)	Natural 12-h control photoperiod as control, extend the photoperiod to 22 h	22/17°C day/night; immature seed harvest and drying	In a glasshouse with a natural variable photoperiod (10–16 h), only 2–3 generations of wheat, barley, chickpea, and canola per year, speed breeding stimulated early flowering and seed set and shortened breeding cycles, 4–6 generations of these crops per year.	6 generations per year	Watson et al., 2018
Durum wheat (*T. durum*)					
Barley (*Hordeum vulgare*)					
Chickpea (*Cicer arietinum*)					
Pea (*Pisum sativum*)					
Canola (*Brassica napus*)				4 generations per year	
Hot pepper (*Capsicum* spp. cv. “Xiangyan55”)	14-h, 16-h, and 18-h photoperiod	420 µmol·m^−2^·s^−1^	The 20-h photoperiod reduced flowering time, and the shortest day from sowing to flowering was 37 days. The breaker stage was reached at 82 DAS (days after sowing), and the red ripening stage was reached at 86 DAS.	4 generations per year	Liu et al., 2022
Hot pepper (*Capsicum* spp. cv. “Xiangla712’)			Plants under the 20-h photoperiod reached the flowering stage at 43 DAS, the breaker stage at 90 DAS, and the red ripened stage at 95 DAS.	4 generations per year	
Lentil (*Lens culinaris* Medik.) cross-derived F_2_ lines	A conventional control treatment under a glasshouse of 10-h to 12-h photoperiod as control, extended photoperiod treatment of 22 h in a growth chamber	Using a single seed descent method (SSD) under the extended photoperiod treatment	Days to maturity under extended photoperiod method was 84 days while glasshouse-based conventional method was 172 days.	3–4 generations per year	Omar, 2020
Wild accessions of *L. orientalis*			Days to maturity under an extended photoperiod method was 115 days while the glasshouse-based conventional method was 225 days.		
Moroccan varieties lentil (*Lens culinaris* “Bakria” and “L24”)	22-h, 18-h, and 14-h photoperiod	Temperature of 23–26°C light/12–18°C dark	A photoperiod of 18 h as a balance between 22 h and 14 h was more optimal for speeding up the breeding cycles.	Not available	Mitache et al., 2023
Chickpea (*Cicer arietinum* “Bouchra” and “Local Chaouia”)					
Faba bean (*Vicia faba* L. “Hiba” and “Loubab”)					
Strawberry (*Fragaria × ananassa* cv. “Albion”)	Photoperiodic treatment of 24-h and 18-h continuous light and natural daylight	Using incandescent light as the predominant light source	Flowering response for “Albion” established a similar degree of sensitivity to photoperiod.	Not available	Sidhu et al., 2022
	Supplemented for long-day (LD; 24 h) and short-day (SD; 10 h) photoperiods	Using light-emitting diodes (LEDs) as the light source	LD photoperiod exposure of diverse light combinations significantly promoted the inflorescences and flower buds inside the crown.	Not available	

### 4.2 Light quality

Light quality, as another critical signal besides photoperiod, has broad effects on plant growth and developmental processes, including seed germination, photomorphogenesis, and flowering (Cerdán and Chory, 2003; Kami et al., 2010; Jones, 2018; Paradiso and Proietti, 2022). Rapid advances in LEDs make it possible to obtain vegetal material with desired traits through the application of specific light wavelengths and accelerate flowering and seeding to advance to the next breeding generation as quickly as possible (Brazaityte et al., 2016; Al Murad et al., 2021). Using optimal light quality could accelerate photosynthesis and flowering and shorten the length of the breeding cycle (**Table 3**).

**Table 3:** Opportunity of light quality on crop speed breeding in plant factories.

Plant species	Light quality	Other experimental details	Influence on speed breeding generations		Reference
			Effects	Generations/year	
Petunia (*Petunia hybrida*)	White light Monochromatic red light Monochromatic blue lights	70 or 150 µmol·m^−2^·s^−1^	Blue lights hasten plant flowering, whereas red light did not display flowering promotion effect.	Not available	Fukuda et al., 2016
Soybean [*Glycine max* (L.) Merr]	1,018 µmol·m^−2^·s^−1^ red (80%) and blue (20%) LED 1,190 µmol·m^−2^·s^−1^ full-spectrum (FS) white light is considered the control	25/18°C, day/night, 12-h photoperiod	RB LED coupled with photothermal conditions can reduce the generation cycle by 56–66 days compared with regular field conditions by 120 days.	5 generations per year	Harrison et al., 2021
*Hippeastrum hybridum* “Red Lion”	Red/blue light ratio of 1:9 (R10B90) and 9:1 (R90B10)	200 µmol·m^−2^·s^−1^, 14-h photoperiod	Higher blue light and low (1/10) red light intensity (R10B90) promoted early flowering and shorted flowering period.	Not available	Wang et al., 2022
Wheat (*Triticum aestivum* L.)	White light (W), white–green light (W:G = 4:1, W4G1), red–green light (R:G = 4:1, R4G1), red–green–blue light (R:G:B = 4:1:1, R4G1B1), red–blue lights (R:B = 3:1, R3B1; R:B = 2:1, R2B1; R:B = 1:1, R1B1; R:B = 1:6, R1B6)	360 µmol·m^−2^·s^−1^, 22-h photoperiod	Higher blue light ratio, such as R2B1 (38.1%), R1B1 (58.7%), and R1B6 (85.8%) delayed flowering time and produced fewer grains. R4G1 induced early flowering time, high yield, and excellent quality, which could be the recommended light environment for indoor wheat cultivation.	Not available	Guo et al., 2022
Tomato (*Solanum lycopersicum* L. cv. Micro-Tom)	Supplementary 100 µmol·m^−2^·s^−1^ red light	Supplementary red light for 12 h per day (6:00−18:00) at the onset of anthesis	Supplementary red light targeted genes that are linked to ripening and promoted the biosynthesis and signaling of ethylene, resulting in the earlier ripening of tomato fruit.	Not available	Zhang et al., 2020
Tomato (*Solanum lycopersicum* L. cv. Micro-Tom)	Supplementary 100 µmol·m^−2^·s^−1^ blue light	Supplementary different blue light frequencies 6 h, 8 h, 10 h, and 12 h with the same intensity at the onset of anthesis	Different frequencies of supplemental blue light accelerated flowering and promote fruit ripening approximately 3–4 days early via promoting ethylene evolution of fruits.	Not available	He et al., 2022
Tomato (*Solanum lycopersicum* L. cv. “Mini Chal”)	Supplement 0.4, 0.6, 0.8 W·m^−2^ UV-A	Basal light: R3B7 [red (R):blue (B) = 30:70], 25/18°C, day/night 50 ± 10% RH, 200 µmol·m^−2^·s^−1^, 12-h photoperiod	UV-A (0.4 W·m^−2^) light intensities promoted faster flowering.	Not available	Kim and Hwang, 2019
Lupin (*Lupinus angustifolius* L.)	R:FR ratio of 5.86, 3.42, 2.89, 2.16, and 1.14	Not available	An R:FR ratio above 3.5 might inhibit flowering while those below 3.5 might induce earlier flowering.	Not available	Croser et al., 2016
Hot pepper (*Capsicum* spp.)	Additional FR light intensity was set to 30, 50, 70, and 90 µmol·m^−2^·s^−1^	Basal light: white:red:blue = 3:2:1, 420 µmol·m^−2^·s^−1^, 12 h photoperiod	Supplementation low-intensity far-red light (30 µmol·m^−2^·s^−1^, R:FR = 2.1) speed up the flowering and significantly accelerated the red ripening of pepper fruit and improved seed germination rates.	4 generations per year	Liu et al., 2022
Winter canola (*Brassica napus* cv. “Darmorbzh”)	Additional FR 500 µmol·m^−2^·s^−1^	22-h light period; 22°C, humidity 70%	Generated visible flower buds at 92 DAG and mature seeds at approximately 125 DAG.	3 generations per year	Song et al., 2022
Spring canola (*Brassica napus* cv. “Westar”)			The life cycle accelerated by 12 days and mature seeds at approximately 55 DAG.	4.5 and 5.5 generations per year	
Semi-winter canola (*Brassica napus* cv. “ZS11”)			The life cycle accelerated by 21 days and mature seeds at approximately 66 DAG.	4.5 and 5.5 generations per year	
Geranium (*Pelargonium × hortorum* L.H.)	Supplement 10 µmol·m^−2^·s^−1^ green light during night interruption	20 ± 1°C, 60 ± 10% RH, 140 ± 20 µmol·m^−2^·s^−1^, 350 ± 50 µmol·m^−2^·s^−1^	Hasten flowering.	Not available	Park et al., 2017
Chinese kale (*Brassica alboglabra*)	Supplement 3 W·m^−2^ (FR-3), 6 W·m^−2^ (FR-6) far-red-light	21 ± 2°C, 55%–60% RH, CO_2_ concentration (400–600 µmol·mol^−1^) Basal light: white LED light (250 µmol·m^−2^·s^−1^ PPFD), 10-h photoperiod.	The budding rate of no-far red light treatment (control group) was only 11.1%, while that of FR-3 and FR-6 reached 30.6% and 45.8%, respectively after 45 days FR supplement. FR accelerated flowering via regulating expression of key genes in the plant circadian rhythm pathway.	Not available	Li et al., 2023
Chinese kale (*Brassica alboglabra*)	Supplement 40 µmol·m^−2^·s^−1^ UVA	Basal light: white 250 µmol·m^−2^·s^−1^, 12-h photoperiod UVA exposure for 6 h/d	Induced faster flowering of Chinese kale than no-UVA treatment.	Not available	Gao et al., 2022
Petunia (*Petunia × hybrida*)	Supplement green light intensity 0, 2, 13, or 25 µmol·m^−2^·s^−1^	Not available	Accelerated flowering of all long-day plants (Petunia, Ageratum, Snapdragon, and Arabidopsis) and delayed flowering of all short-day plants (Chrysanthemum and Marigold).	Not available	Meng and Runkle, 2019
Ageratum (*Ageratum houstonianum*)					
Snapdragon (*Antirrhinum majus*)					
Arabidopsis (*Arabidopsis thaliana*)					
Chrysanthemum (*Chrysanthemum morifolium*)					
Marigold (*Tagetes erecta*)					

Red and blue LEDs have been widely applied in horticulture, since these two spectral regions are regarded as particularly significant to photosynthetic CO_2_ assimilation (Chory, 2010). Both blue and red light are involved in the regulation of different processes through the plant life cycle (including seed germination and vegetative and reproductive growth). Blue light is a predominant signal for flower initiation; it can promote floral induction and flowering by regulating the level of hormones and gene expression associated with flowering (Yoshida et al., 2016; He et al., 2022; Wang et al., 2022; Nie et al., 2023). Inversely, red light has been recorded to promote both vegetative and reproductive growth but hinder flowering (Fukuda et al., 2016; Wang et al., 2022). However, it should be noted that flowering responses to blue and red light need to be further explored in various crop species since the research results often contain contradictory information to date (Giliberto et al., 2005; Monostori et al., 2018; Zhang et al., 2020; He et al., 2022).

Acceleration of flowering was well identified in many crops such as *Arabidopsis thaliana, Hordeum vulgare, Petunia hybrida*, and *Zinnia elegans*, in response to low R:FR ratios (Demotes-Mainard et al., 2016). In general, an approximate R:FR of 1.2, which occurred under natural sunlight, is suitable for plant growth and flowering (Holmes and Smith, 1975). However, the thresholds of R:FR ratio regarding flowering time vary among plant species and varieties, as well as growing conditions. For example, thresholds of R:FR ratio that promote or delay flowering were 3.5 and 5.3 in *Eustoma grandiflora* and lupin, respectively (Yamada et al., 2009; Croser et al., 2016). FR was beneficial for plants to facilitate light interception and tremendously accelerate plant growth and development responsible for the wide application in a controlled environment to induce early flowering and shorten breeding cycles (Liu et al., 2022; Song et al., 2022; Li et al., 2023). Besides R and FR light, blue light, in specific conditions, and some other light (i.e., green and ultraviolet-A) are also constructive for regulating the flowering of a few species such as petunia, tomato, and Chinese kale (Smith et al., 2017; Verdaguer et al., 2017; Kim and Hwang, 2019; Gao et al., 2022). To facilitate the optimal utilization of LEDs in the realm of horticultural crop production within plant factories, a comprehensive comprehension of the effects of light wavelengths on plant performance, encompassing the underlying mechanisms and temporal aspects, becomes imperative.

### 4.3 Light intensity

To date, a considerable number of studies focus on clarifying the impact of light photoperiod and light quality on plants, while those that target plant responses to light intensity are relatively few, even though plants have developed a sophisticated mechanism to alter their morphological features in response to varying intensity. Generally, under the light saturation point, increasing light intensity and the photosynthesis rate, measured as photosynthetic oxygen evolution, increases linearly with the light, because in this phase, light is the limiting factor (Haliapas et al., 2008; Formighieri, 2015; Oh, 2015). Higher light intensity provides more photosynthetic energy required for the synthesis of flowering-promoting substances, which is conducive to the formation and growth of flower primordium (Wimalasekera, 2019). Thus, reasonable light intensity can promote photosynthesis, allowing more light assimilation substances to be distributed to seeds or fruits, leading to rapid growth, higher biomass, and greater seed production (**Table 4**). Advancements in LED technologies have significantly contributed to achieving optimal light intensity for facilitating plant growth, thereby expediting plant life cycles and enhancing the completion of multiple generations within a shorter time frame (Jähne et al., 2020; Liu et al., 2022). Notably, the augmentation of light intensity, concomitant with escalated energy expenditures, manifests only marginal alterations, necessitating a comprehensive evaluation of the balance between advantages and associated costs.

**Table 4:** Effect of light intensity on crop speed breeding.

Plant species	Light intensity	Other experimental details	Influence on speed breeding generations		Reference
			Effects	Generations/year	
Petunias (Petunia *×* hybrida)	Low (L) light: 40 µmol·m^−2^·s^−1^ Medium (M) light: 120 µmol·m^−2^·s^−1^ High (H) light: 360 µmol·m^−2^·s^−1^	HPS; 16 h·d^−1^ photoperiod	Flower bud formation was achieved at the end of the 4th, 5th, and 6th week after H, M, and L, respectively.	Not available	Haliapas et al., 2008
*Eustoma grandiflorum* cv. “Nail Peach Neo”	Light intensity increased from 100 to 400 µmol·m^−2^·s^−1^	10 h·d^−1^ photoperiod	Flowering time was shortened by 7 to 10 days.	Not available	Oh, 2015
Soybean (*Glycine max*)	480, 720, 960, 1,160, and 1,511 µmol·m^−2^·s^−1^	10 h·d^−1^ photoperiod	Above 1,000 µmol·m^−2^·s^−1^ flowering at ~21.8 days after planting while 500–900 µmol·m^−2^·s^−1^ at 23.9 days. Thus, ~500 µmol·m^−2^·s^−1^ should suffice to achieve fast generation times on a moderate budget.	Generation cycle by 75 days	Jähne et al., 2020
Hot pepper (*Capsicum* spp.) cv. “Xiangyan55”	240, 300, 360, and 420 µmol·m^−2^·s^−1^	12 h·d^−1^ photoperiod	300 to 360 µmol·m^−2^·s^−1^ reduced flowering time. Completely red earliest under 360 µmol·m^−2^·s^−1^, only 86 days from sowing to full maturity.	Generation cycle by 86 days 4 generations per year	Liu et al., 2022
Hot pepper (*Capsicum* spp.) cv. “Xiangla712”			The earliest to reach each physiological stage was under 420 µmol·m^−2^·s^−1^, which reached the flowering stage at 43 days, the breaker stage at 83 days, and the red ripened stage at 86 days.		

### 4.4 Temperature

Apart from the light environment, temperature is particularly important to manipulate plant reproductive development and accelerate plant flowering. Each species has a specific temperature regime (maximum and minimum temperatures) for growth and development. The temperature range conducive to the germination of most crops typically falls within 12°C to 30°C, while the optimal temperature range for growth, flowering, and seed setting is generally observed between 25°C and 30°C (Hatfield and Prueger, 2015). Within the temperature range that does not cause heat stress to plants, higher temperature might facilitate vegetative growth while lower temperature is more suitable for grain production in the reproductive stages (Hickey et al., 2019). Optimum temperature is beneficial to accelerate photosynthesis and flowering, coupled with early seed harvest to shorten the breeding cycle, such as the SB approach applied in peanut, chickpea, lupin, lentil, pea, soybean, and faba bean (O’Connor et al., 2013; Croser et al., 2016) (**Table 5**). Notably, some crops must undergo a period of low temperature to transition from vegetative growth to reproductive growth, that is, vernalization. For instance, to achieve typical flower initiation and formation and accelerate plant growth, winter wheat requires long exposure to low temperatures (Cha et al., 2022). Hence, overcoming the rapid vernalization of winter crops stands as a pivotal technological advancement for expediting breeding processes in plant factories.

**Table 5:** Effect of temperature on crop speed breeding.

Plant species	Temperature	Other experimental details	Influence on speed breeding generations		Reference
			Effects	Generations/year	
Sorghum (*Sorghum bicolor* L. cv.) “Moench”	31.9/21.0°C (day/night) 32.8/21.0°C (day/night) 36.1/21.0°C (day/night) 38.0/21.0°C (day/night)	14 h·d^−1^ photoperiod, 600 µmol·m^−2^·s^−1^	Average seed set percentage decreased significantly from 80% and 69% to 59% and 31% as maximum temperature increased across the four temperature regimes.	Not available	Singh et al., 2015
Grain amaranths (*Amaranthus* spp.)	35°C/30°C, 16-h day length 30°C/25°C, 8-h day length	Single-nucleotide polymorphism (SNP) markers	Maintained at 30°C induced early flowering 4 weeks after planting and allowing amaranth to complete one breeding generation for 2 months, while approximately 6 months are needed to take in the field.	6 generations per year	Stetter et al., 2016
Early flowering pea cultivars (*Pisum sativum* L. cv.) “PBA Twilight”	20/18°C (day/night) 24/20°C (day/night)	13–14 h·d^−1^ photoperiod, 600 µmol·m^−2^·s^−1^, *in vivo*/*in vitro* SSD	Increasing temperature resulting in early flowering by 6.58–10 days. Growing plants under optimized temperature 24/20°C (day/night) allowed up to 2.5 times faster floral onset compared to field conditions and up to 1.7 times compared to the conventional SSD system across the entire range of pea genotypes tested.	Not available	Ribalta et al., 2017
Mid-flowering pea cultivars (*Pisum sativum* L. cv.) “PBA Pearl”					
Late-flowering pea cultivars (*Pisum sativum* L. cv.) “Kaspa”					

### 4.5 CO_2_ concentrations

Carbon dioxide (CO_2_) serves as the principal substrate for both photosynthesis and photorespiration in higher plants, thereby playing a pivotal role as a predominant greenhouse gas that regulates crop growth and development. Plant growth and flowering exhibit sensitivity to varying CO_2_ levels, whose response depends on the species and environmental conditions (McGrath and Lobell, 2013). Overall, elevated concentrations of CO_2_ have generally facilitated expedited plant growth and hastened the transition from vegetative to reproductive stages across most species (Jagadish et al., 2016). This effect is particularly pronounced among C3 plants, which exhibit greater sensitivity to heightened CO_2_ levels compared to C4 plants. Supplemental CO_2_ or the remaining higher CO_2_ concentration in growth chambers contributes to the reduction in the days to flowering of rice and Arabidopsis (Ohnishi et al., 2011; Li et al., 2014; Tanaka et al., 2016) (**Table 6**). Regulation of CO_2_ level often combines with other optimal light, temperature, and innovative breeding technologies (i.e., embryo rescue, biotron breeding system, and using immature seeds) to shorten the breeding cycle length in the controlled environment (Tanaka et al., 2016; Nagatoshi and Fujita, 2019).

**Table 6:** Effect of CO_2_ concentrations on crop speed breeding.

Plant species	CO_2_ concentrations	Other experimental details	Influence on speed breeding generations		Reference
			Effects	Generations/year	
*Arabidopsis thaliana*	Ambient CO_2_ (380 ppm), low CO_2_ (100 ppm)	8 h·d^−1^ photoperiod, 150 µmol·m^−2^·s^−1^, 70% relative humidity, 21°C	Flowering time was delayed by 7 days under low CO_2_ (100 ppm) compared to normal CO_2_ (380 ppm) conditions	Not available	Li et al., 2014
Rice (*Oryza sativa* cv. Nipponbare)	Supplemental 475 ppm CO_2_	30°C/25°C, light/dark, 14 h·d^−1^ photoperiod, regulation of CO_2_ level, embryo rescue, removal of tillers	Mean flowering time was 6 d earlier under regulated CO_2_ conditions compared with no CO_2_ regulation. Shorten the generation time of cv. Nipponbare plants to approximately 2 months.	6 generations per year	Ohnishi et al., 2011
Rice (*Oryza sativa* L. cv. “Nipponbare”)	Supplemental 600 ppm CO_2_	27°C/25°C, light/dark, 14 h·d^−1^ photoperiod, 230 µmol·m^−2^·s^−1^. Biotron breeding system (BBS), without requiring tiller removal	Higher CO_2_ treatment shortened the days to heading by 2.7 days and 9.3 days in “Nipponbare” and “Yamadawara”, respectively, and shortened the generation cycle of rice to less than 90 days, enabling 4 generations of rice to be obtained per annum, instead of only 1–2 generations per year in the field and/or greenhouse.	4 generations per year	Tanaka et al., 2016
Rice (*Oryza sativa* L. cv. “Yamadawara”)					
Soybean (*Glycine max* L. cv. “Merr”)	Supplemental CO_2_ >400 ppm	14 h·d^−1^ photoperiod, 27°C/25°C, light/dark, use of immature seeds	CO_2_ supplementation promoted the number of high-quality flowers and crossing efficiency, but did not affect the days to flowering. Utilizes CO_2_ in combination with optimal light shortened the breeding cycle length from 102–132 d to 70 d, which permits 4–5 generations a year compared to 1–2 generations on the field.	4–5 generations per year	Nagatoshi and Fujita, 2019

### 4.6 Density of plant populations

Planting density is an important agronomic management, and optimization of density will determine the productivity and economic returns of plant factory with artificial lighting (PFAL). Higher planting density promotes shoot positioned upward and leads to the elongation of stems and leaves to outcompete its neighbors for enough sunlight, ultimately accelerating the transition from a vegetative stage to a reproductive stage and triggering early flowering and maturation (Warnasooriya and Brutnell, 2014). Additionally, higher cultivation density provides more sufficient leaf area to absorb sunlight efficiently or improve symbiotic nitrogen fixation by root nodules, thus benefiting early flowering or improving grain yield (Abubaker, 2008; Makoi et al., 2009; Biosci et al., 2013; Rahmanet, 2019; Asemanrafat and Honar, 2023) (**Table 7**). In contrast, the greater availability of fertilizers per plant due to low plant density results in more vigorous vegetative growth and late flowering (Abubaker, 2008). However, biomass increase was not linear with the planting density. Excessive planting density results in an intra-specific competition for space, nutrients, and light, causing the source–sink ratio to be not reasonable and ultimately hinder plant growth and development (Khan et al., 2017).

**Table 7:** Effect of plant density on crop speed breeding.

Plant species	Density of plant populations	Other experimental details	Influence on speed breeding generations		Reference
			Effects	Generations/year	
Bean (*Phaseolus vulgaris* L.)	Planting densities (10*30 cm, 20*30 cm, 30*30 cm, 40*30 cm, 50*30 cm, 60*30 cm)	8 h·d^−1^ photoperiod, 150 µmol·m^−2^·s^−1^, 70% relative humidity, 21°C	Planting density of 20*30 cm results in the highest yields compared to other planting densities. Greater availability of fertilizers per plant due to low plant density results in more vigorous vegetative growth and late flowering.	Not available	Abubaker, 2008
Cowpea (*Vigna unguiculata* L. cv. “Walp”)	High plant density: row-to-row spacing of 60 cm, and plant-to-plant distance of 20 cm. Low plant density: row-to-row spacing of 90 cm, and plant-to-plant distance of 40 cm	Not available	The earliest flowering date was observed in high-density cropping that improved symbiotic nitrogen fixation by root nodules in cowpea.	Not available	Makoi et al., 2009
Spotted bean (*Phaseolus vulgaris* L.)	15, 25, 35, and 45 plants/m^2^	Not available	The maximum grain yield was related to the density of 45 plants/m^2^ while the minimum yield was in the density of 25 plants/m^2^; grain yield increased with plant density increasing might be attributed to sub branches, which are placed at the lower part of the plant, exerting obvious influence on biological yield increase.	Not available	Biosci et al., 2013
Rice (*Oryza sativa* L.)	5 cm × 5 cm spacing (over 4,000,000 plants/hectare), 20 cm × 15 cm spacing (300,000 plants/hectare)	Not available	A high density triggered early flowering, and shortened the growth duration of rice plants compared to the traditional plant density.	Not available	Rahmanet, 2019
Red beans (*Phaseolus vulgaris* L. cv. Akhtar)	Row spacing of 5 cm, 10 cm, and 15 cm	Not available	Higher density (row spacing of 5 cm, 66 plants/m^2^) provides more sufficient leaf area to absorb sunlight efficiently and achieved the highest yield when compared to the row spacing of 10 and 15 cm, whose plant density was 33 and 22 plants/m^2^, respectively.	Not available	Asemanrafat and Honar, 2023

### 4.7 Nutrition and hormones

Flowering is an important phase in the life cycle of plants, which is profoundly impacted by numerous exogenous and endogenous factors. Five different pathways, namely, photoperiod, vernalization, gibberellin acids (GAs), autonomous, and endogenous pathways, have been reported to regulate flowering time (Mouradov et al., 2002; Blümel et al., 2015). Five major types of plant hormones, namely, auxin, gibberellin, cytokinin, ethylene, and abscisic acid, are important to induce flowering and seeding as well as germination of immature seed *in vitro* working together or independently (Bermejo et al., 2016). Exogenous application of GAs accelerated flowering in wild-type Arabidopsis (Blázquez et al., 2015). Additionally, cytokinins (5.7 μM) in conjunction with auxin (2.3 μM) led to the highest rate of flowering (100%) and seeding (91%) with the shortest flowering time (32 d) in lentil and faba bean; this combination promoted plant flowering and seeding rates by 4.9 d in advance (Mobini et al., 2015). Moreover, applying 10^–5^ M of 6-benzylaminopurine (BAP) accelerated flowering in faba bean, with the generation time reduced by 20–24 days (Mobini et al., 2020). Despite the widespread demonstration of phytohormone-mediated flowering regulation potential, plant hormone application in breeding is constrained by several limitations. On the one hand, plant hormones exhibit intricate and extensive regulatory effects on growth and development, potentially leading to unanticipated physiological and morphological changes during the regulation of flowering. This complexity poses challenges for the precise control of hormone-mediated regulation in breeding and may result in unforeseen side effects. On the other hand, phytohormone-mediated flowering regulation is heavily influenced by environmental factors such as light, temperature, and water availability, leading to inconsistent effects in varying growth environments and limiting its stability and reliability in breeding practice. Additionally, the impact of hormones on genetic stability and safety further discourages their widespread use in breeding practices.

Among essential nutrients, nitrogen (N), phosphorus (P), and potassium (K) are the most prominent nutrients required in greater amounts for the proper growth and development of crops (Ye et al., 2019). Nitrogen (N) as a most abundant essential nutrient plays critical roles in the process of flowering. The influence of N on flowering time greatly varies with different N forms and concentrations. In general, flowering is often accelerated by low N concentration, because limited N concentration enhances the export rate of assimilates from vegetative tissues to grains during the post-anthesis period. In contrast, excessive ammonium N supply delayed flowering by hindering N transport in the phloem at the flowering stage and decreased the efficiency of N remobilization (Marín et al., 2011; Zhang et al., 2021). A hypothetical U-shaped flowering curve was proposed, in that an appropriate concentration of nitrate promotes flowering, while both limited and excessive nitrate supply result in late flowering (Lin and Tsay, 2017). Phosphorus (P) fertilizer is a crucial nutrient for normal plant growth, development and yield, which is taken up by plant roots as inorganic phosphate (Pi) transporters (Muchhal et al., 1996). The flowering of rice and Arabidopsis grown under P deficiency conditions was prolonged (Kant et al., 2011; Jiaying et al., 2022). A longer time to flower was shown in low P levels, which might have contributed to the low gibberellin levels, where P level was positively correlated with gibberellin levels (Jiang et al., 2007). Notably, an antagonistic crosstalk was observed on the flowering between NO_3_^−^ and Pi, in which low NO_3_^−^ caused earlier flowering while low Pi postponed flowering time in Arabidopsis (Linkohr et al., 2002; Kant et al., 2011). Otherwise, the application of K fertilization led to flowering 1–3 days earlier in rice, and flowering time was faster as the application rate of K fertilizer increased (Ye et al., 2019). Thus, a robust and intelligent water and fertilizer control system needs to be created based on the demand for water and fertilizer for horticultural crops’ SB in a plant factory.

## 5 Challenges and perspectives

Remarkable breeding approaches in the last few decades have allowed researchers to shorten the breeding cycles, but they were limited in scale and cost and insufficient to cope with the ever-increasing population and radical changes under adverse environmental conditions. Integrating SB in plant factories with biology, information science, and breeding science to accelerate rapid growth, development, flowering, and seeding and establishing a rapid crop breeding technology system are a promising option to shorten the breeding cycle. However, every coin has two sides; thus, SB in plant factories has its advantages and limitations.

### 5.1 Challenges of speed breeding in plant factories

Tremendous advancements have been achieved in smart breeding recently, which is driven by big data, artificial intelligence, and integrated genomic–environmental prediction, but considerable challenges remain to be addressed for SB in plant factories. Firstly, the high cost of energy and considerable investment in setup and operation are major drawbacks of an artificially lighted plant factory. The electricity cost typically accounts for 25% of the total production cost (Kozai and Niu, 2015b), with the lighting, air conditioning, and other information control system depending on electricity. Advances in LED technology have provided more efficient power usage and reduced heat than other lighting types. Investing in solar panels is a good strategy to offset the erratic electricity supply in SB in plant factories.

Secondly, there is a lack of rapid vernalization techniques for long-day crops, and determining the short photoperiod for short-day crops remains challenging. Therefore, longer time needs to be taken to obtain the optimal environmental conditions (light intensity, light quality, temperature, etc.) for the rapid generation advancement of target crops.

The expression of the same phenotypes in both controlled environments and field conditions because of genotype × environment interactions is considered a challenge in SB. Thus, smart breeding driven by big data, artificial intelligence, and integrated genomic–enviromic prediction is an area that requires further enhancement in the future. Although phenotyping technology and platform have been widely applied in controlled environments for crop genetic improvement and breeding, compared with genomics, transcriptomics, and proteomics, phenomics is lagging behind. At present, a lack of *in vivo* dynamic detection technology for crop phenotypes, a poor ability to obtain high-throughput crop phenotypes, and the unclear relationship between genotype, phenotype, and environmental metrics limit the acquisition and analysis of crop phenotypes in plant factories. Therefore, with the targeted goal of horticultural crop breeding, a collaborative breeding framework of various branches of science is paramount to speed up breeding to accelerate crop improvement for a rapidly expanding global population.

### 5.2 Smart breeding: speed breeding in plant factories integrates with innovative breeding technologies

In the short-term or long-term future, SB in plant factories where the light environment (light quality, light intensity, and photoperiod), temperature, and nutrient are modulated accurately and efficiently holds promise to relieve the long-standing problem of lengthy crop breeding cycles. Notably, processes as diverse as seed germination, flower bud differentiation, flowering and fruiting, and seed development are closely regulated by light, temperature, phytohormone, and nutrition, and accelerating the above process is conducive to shortening the growth cycle of crops using the conditions provided by the smart system in plant factories.

Interdisciplinary strategies consisting of plant breeding, genome sequencing, genomic selection (GS), and CRISPR-based genome editing help plant breeders improve existing crops and develop new crops with good agronomic traits by minimizing time, cost, and space and maximizing resource efficiency. SB in plant factories is a promising approach that allows breeders to rapidly develop lines with the desired trait by advancing generations quickly within a controlled environment. Numerous steps are required for SB to effectively work in plant factories. Optimization of light formula and nutrient solution formula for species specificity and the establishment of rapid generation advancement technology within plant factories were the cornerstone for SB in plant factories. Moreover, overcoming physiological seed dormancy, combined with early seed harvest, is critical to reduce the length of the breeding cycle.

Phenotypic performance or target trait observation was the first step in plant breeding, and identifying desirable traits for selective breeding has become the crucial problem restricting crop breeding owing to the presence of masking. Recent advances in *in vivo* imaging, hyperspectral imaging, and graphic and spectral analysis technology, which dramatically help to measure crop phenotypic traits rapidly and nondestructively in controlled environments, make high-throughput phenotyping a powerful approach to solving future crop breeding problems.

**Figure 2:**
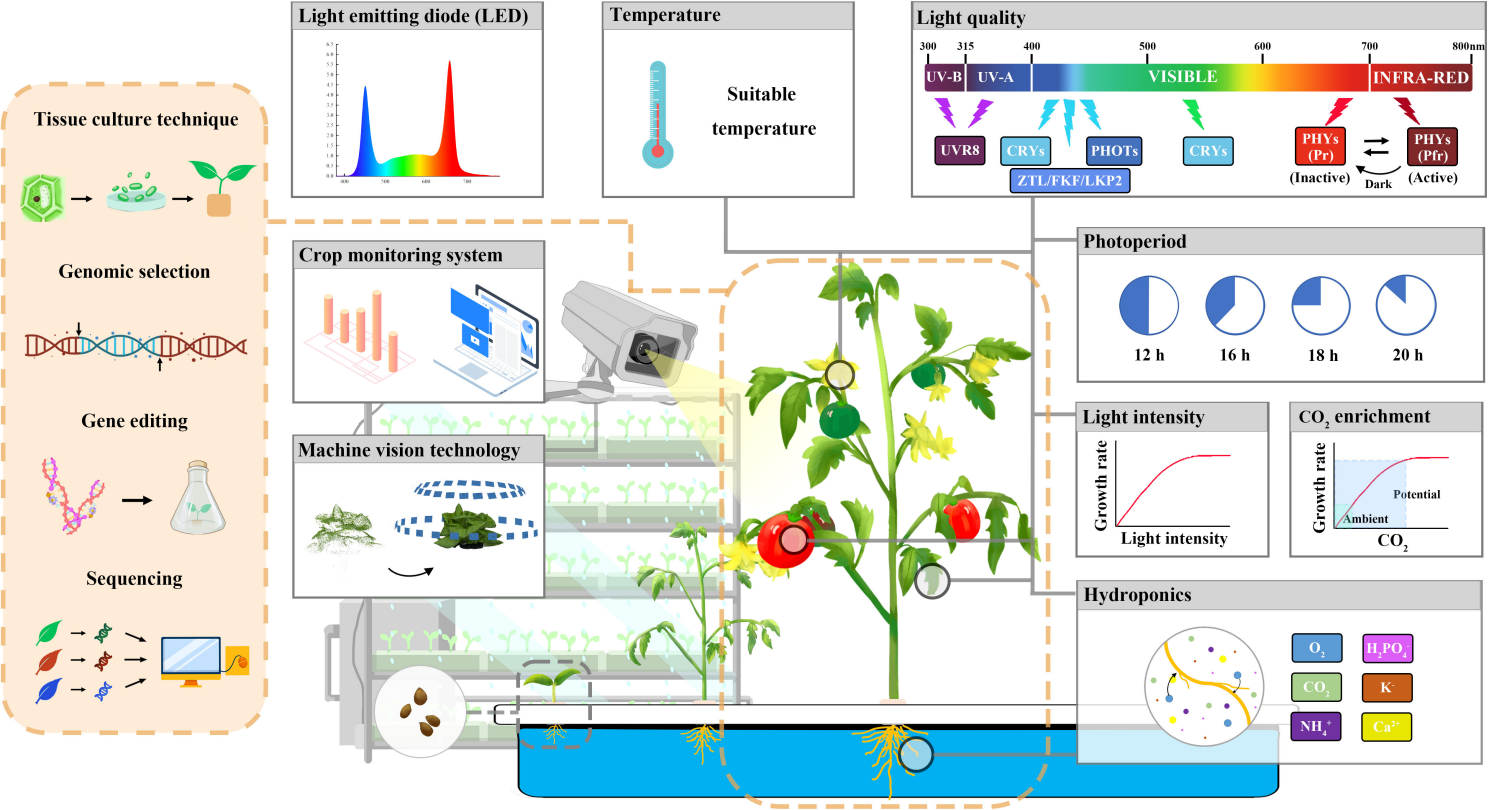
Vegetable speed breeding in a plant factory.

Overall, the integration of innovative breeding technologies such as embryo rescue and immature seed harvesting, **TILLING,** gene editing, high-throughput phenotyping and genotyping, genomic selection, marker-assisted selection (MAS), machine learning, deep learning, and omics technologies to form a smart breeding platform offers an attractive opportunity to promote the transformation of breeding technology from the traditional “experience breeding” to a directional and more efficient “precision breeding” (**Figure 2**).

## Author contributions

RH: Conceptualization, Methodology, Writing – original draft, Writing – review & editing. JJ: Conceptualization, Data curation, Validation, Writing – original draft. KL: Conceptualization, Methodology, Writing – original draft. JS: Conceptualization, Supervision, Validation, Writing – review & editing. XL: Conceptualization, Methodology, Writing – original draft. YL: Conceptualization, Methodology, Writing – original draft. SZ: Data curation, Investigation, Methodology, Writing – original draft. MZ: Conceptualization, Methodology, Writing – review & editing. YH: Conceptualization, Methodology, Writing – original draft. HL: Conceptualization, Funding acquisition, Investigation, Methodology, Project administration, Resources, Supervision, Writing – review & editing.
